# Highly effective ashless and non-corrosive dimercaptobenzothiadiazole as multifunctional lubricant additives in naphthenic base oil†

**DOI:** 10.1039/d3ra05692a

**Published:** 2023-10-19

**Authors:** Chiu Ling Ong, Yew Chong Lai, Thorsten Heidelberg, Wai Kit Tang, Vannajan Sanghiran Lee, Nader Ghaffari Khaligh, Joon Ching Juan

**Affiliations:** a Nanotechnology and Catalysis Research Centre, Level 3, Block A, Institute for Advanced Studies, University of Malaya 50603 Kuala Lumpur Malaysia jcjuan@um.edu.my; b Oleon Port Klang Sdn. Bhd. 57, Jln Sungai Pinang 4/3, Taman Perindustrian Pulau Indah 42920 Pelabuhan Klang Selangor Malaysia; c Department of Chemistry, Faculty of Science, University of Malaya 50603 Kuala Lumpur Malaysia; d Department of Chemistry, Centre of Theoretical and Computational Physics (CTCP), Faculty of Science, University of Malaya 50603 Kuala Lumpur Malaysia; e Faculty of Engineering, Technology and Built Environment, UCSI University Cheras 56000 Kuala Lumpur Malaysia

## Abstract

The conventional medium chain chlorinated paraffin (MCCP) and zinc dialkyl dithiophosphate (ZDDP) additives have greatly enhanced the extreme pressure (EP) and anti-wear (AW) performance of the metalworking fluids. However, chlorine- and zinc-containing additives are restricted in use due to eco-toxicity issue. Herein, ashless and non-corrosive dimercaptobenzothiadiazole derivatives, namely bis-2,5-benzylsulfanyl-[1,3,4]thiadiazole (BBST) and bis-2,5-octylsulfanyl-[1,3,4]thiadiazole (BOST) consist of three sulfur atoms have been synthesized and evaluated. The performance of BBST shows a weld load (*P*_D_) of 3089 N and AW value of 5 mm^2^, which represents an improvement of 3.1 and 7.4 folds over naphthenic base oil (NBO). In addition, BBST also outperformed BOST, MCCP, and ZDDP in terms of its weld load and AW properties. Based on XPS analysis and molecular electrostatic potential maps (MEPS), BBST exhibits superior tribology performance due to the interaction between the sulfur (S), nitrogen (N), and π-electrons of the benzene ring with the metal surface. The formation of FeS, Fe_2_O_3_ and Fe⋯N coordinate bonds contributes to the creation of an excellent tribofilm.

## Abstract 2

An ashless and non-corrosive bis-2,5-benzylsulfanyl-[1,3,4]thiadiazole shows excellent extreme pressure and anti-wear performance. This is due to the formation of FeS, Fe_2_O_3_, and Fe⋯N coordinate bonds, which contribute to the creation of an excellent tribofilm.

## Graphical abstract

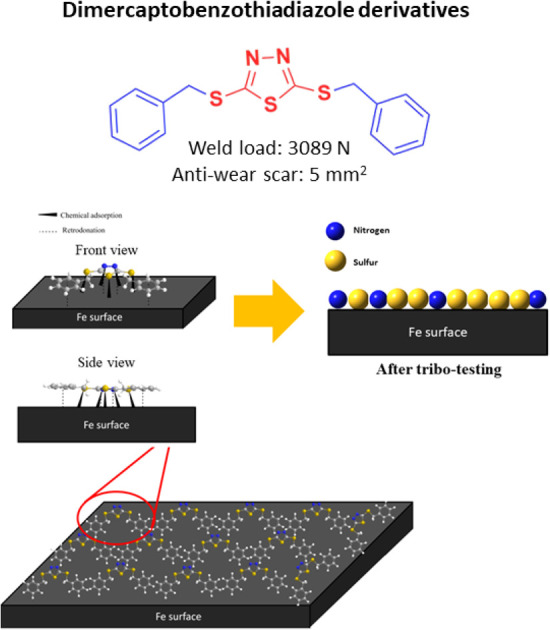

## 1. Introduction

The efficient operation of industrial machinery and equipment plays a significant role in minimizing energy consumption and enabling high productivity. Modern technology has forced the mechanical equipment to work under more harsh operation conditions, such as heavy loads, high temperatures, and high-speed working conditions.^1^ Nevertheless, the long-term operation of machinery and equipment under such operation conditions has resulted in damage, downtimes, and reduced service life of system components due to wear and friction, which in turn affects industrial production. About 20% of the global energy consumption is used to reduce friction, and another 3% is used to replace equipment due to wear.^2^

Metalworking fluids (MWFs) are commonly employed in industrial machining to cool and lubricate the contact region between metal workpiece.^3^ The extreme pressure (EP) and anti-wear (AW) additives are the main components in MWFs. The EP and AW additives interact thermo-chemically and tribo-chemically with the metallic surface, generating a tribo-film that shields the metal surface from further wear and welding.^4,5^ The commonly used MWFs EP and AW additives are chlorine-, phosphorus-, and sulfur-based additives.^6^ Nonetheless, chlorine-based additives are being discussed to be added as the restricted in use by the REACH (Registration, Evaluation, Authorization and Restriction of Chemicals) to reduce chemical eco-toxicity of consumer products,^7,8^ while the phosphorus-based additives have lower EP performance than that of chlorine- and sulfur-based additives, respectively.^6^

Presently, organo-sulfur is the most vital lubricant additive owing to its excellent EP and AW performance.^9^ Moreover, sulfur element is also greener than phosphorus and chlorine elements.^10,11^ Among the variety of sulfur-containing compounds, organo-sulfides, or polysulfides, including sulfurized isobutene, dialkylpentasulfide, and di(iso-butyl) polysulfide have been commercialized as EP lubricant additives owing to their excellent load-carrying capacity. Their weld load performance is up to 7000 N because of high sulfur content (*n* = 5). Nevertheless, an excessive amount of sulfur content can lead to significant problems with metal corrosion and increased wear rate on the metal surface. This can ultimately result in decreased AW performance.^12,13^ In addition, polysulfides additives are also highly corrosive to the copper-based alloy. It was reported that the number of sulfur exceed three atoms a molecule will potentially induce corrosion.^14^ Therefore, the development of ashless, non-corrosive, high EP, and AW sulfur-based additives has attracted the interest of researchers.

Heterocyclic organic compounds, including derivatives of dimercaptobenzothiadiazole, have been identified as effective additives for lubrication. They offer excellent extreme pressure (EP) and anti-corrosion properties as well as thermal stability when used in polar base oils like rapeseed oil and trimethylolpropane trioleate.^15–18^ Besides, the compact molecular structure of dimercaptobenzothiadiazole with rich sulfur and nitrogen atoms can easily interact with the metal surface to generate a stable adsorption tribo-film.^19^ Moreover, the nitrogen elements also improve the biodegradability of friction modifiers and provide sufficient nutrients to microorganisms. Dimercaptobenzothiadiazole is also “ash-free” and “phosphorus-free” which meets the environmental protection regulations.^20,21^ Dimercaptobenzohiadiazole derivatives with three sulfur atoms are highly effective in EP performance. However, the high sulfur content in these compounds can also result in metal corrosion.^22–26^ Additionally, the dimercaptobenzothiadiazole derivatives also shows poor solubility in non-polar base oils, which limits their application as lubricant additives.^17,24^

The polarity of the base oil is a crucial factor in determining the thickness and growth rate of the tribofilm layer created by the additives.^27^ When mixed with non-polar base oils, additives produce a thicker tribofilm due to the lower affinity of base oil molecules to react with the metal surface. This results in increased affinity of additive molecules to react with the metal surface and create a protective tribofilm. Additionally, the compatibility between the base oil and additive is crucial for improving lubrication performance.^28^ An additive with good solubilty can increase the absorptivity of additives on the metal surface, leading to better tribological performance. Since naphthenic base oil (NBO) is non-polar, low aromatic content, high viscosity, better-emulsified ability, low pour point, and compatible with most additives,^29^ it is commonly used as MWFs base stock.

Our previous work^30^ found that disulfide functionalized by alkyl and benzyl groups are completely soluble in NBO and non-corrosive (grade 1a). Besides, the EP and AW performance of disulfide derivatives was also better than MCCP and ZDDP, respectively. Thus, the tribology performance of alkyl- and benzyl-substituted dimercaptobenzothiadiazole derivatives on NBO are worth investigating due to different characteristics. The exploration between the chemical structure and solubility, EP, AW, and corrosiveness performance is necessary to provide the basic understanding.

In this work, a more environmentally friendly and ashless dimercaptobenzothiadiazole additives, namely bis-2,5-benzylsulfanyl-[1,3,4]thiadiazole (BBST) and bis-2,5-octylsulfanyl-[1,3,4]thiadiazole (BOST) were synthesized and used as multifunctional lubricant additives in the NBO. The solubility, corrosiveness, EP, and AW performance of the dimercaptobenzothiadiazole derivatives in NBO were studied.

## 2. Experimental

### 2.1 Materials

2,5-Dimercapto-1,3,4-thiadiazole (analytical reagent, ≥98%), 1-chlorooctane (analytical reagent, ≥99%), benzyl chloride (analytical reagent, ≥99%), anhydrous sodium hydroxide (reagent grade, ≥98%), ethyl acetate (ACS reagent, ≥99.5%), ethanol (ACS reagent, ≥99.5%), and anhydrous magnesium sulfate (ReagentPlus, ≥99.5%) were purchased from Sigma-Aldrich Co. Ltd, which were used as received without further purification. The NBO, zinc dialkyl dithiophosphate (ZDDP), and medium chain chlorinated paraffin (MCCP) were obtained from Oleon Sdn. Bhd. The physical properties of NBO, MCCP, and ZDDP were similar to our previous studies.^30^

### 2.2 Synthesis of dimercaptobenzothiadiazole derivatives

**Scheme 1:**
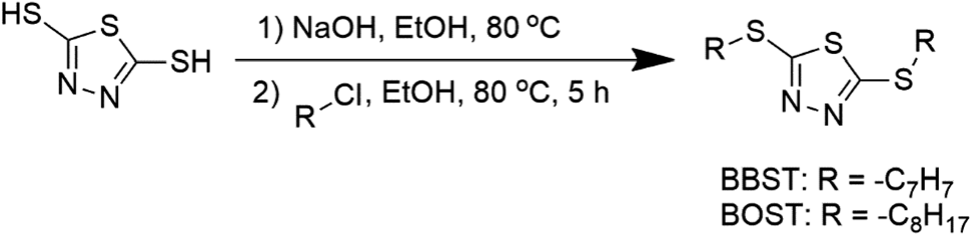
The synthetic route of additives.

BBST and BOST were prepared with modification according to the previous procedure.^31^ 2,5-Dimercapto-1,3,4-thiadiazole (40 mmol) and NaOH (80 mmol) were mixed and stirred in 50 mL ethanol (purity ≥99.5%) at 80 °C until the mixture became a colorless solution. The organic halides (80 mmol) were added and stirred constantly. After the reaction had finished, the mixture was extracted twice using ethyl acetate and water. The resulting solvent layer was then collected and subjected to drying using magnesium sulfate. The next step was to concentrate the solution using a rotavap evaporator, which yielded the desired products. The synthetic pathway for the additives is shown in Scheme 1.

The chemical structure of the synthesized dimercaptobenzothiadiazole additives was confirmed by nuclear magnetic resonance (Brüker Avance III, 600 MHz), Agilent 6560 iFunnel Q-TOF LC-MS, and Agilent 6890N GC-MS. The spectra data were provided in ESI data Fig. S1 and S2.†

### 2.3 Properties testing

The detail of the following test and evaluation were also carried out based on our previous method.^30^ The stability and solubility of BBST and BOST were evaluated with visual observation. The density and viscosity of NBO, 10 wt% BBST, and 10 wt% BOST were evaluated by using a Anton Paar density meter (DMA 5000) and Brookfield NDJ-5S Viscometer. Thermogravimetric analysis (TGA) was evaluated according to ASTM E2550-11 using a TA Instruments TGA-2950 instrument. The corrosion grade of the additives was evaluated with ASTM D130.

### 2.4 Tribological and worn surface analysis

The AW and EP performance of the additives were evaluated according to previous method.^30^ Prior surface analysis, the worn surfaces were cleaned using ethanol in an ultrasonic bath for 15 min (3 times). The morphology and elemental composition of the wear scars were then analyzed using SEM-EDS. XPS analysis (PHI Quantera Model) was also performed to determine the chemical states of the typical elements present. The Al Kα was used as the exciting source, with a pass energy of 1486.6 eV. The reference point for the analysis was the binding energy of C 1s at 284.8 eV.

### 2.5 Computational details

The geometries of BBST and BOST were optimized using the Gaussian 09 software package at the M062X/6-311+g(2d,2p) level of theory.^32^ The empirical dispersion (GD3) environment was used during the structural optimization. A pruner super-fine integral grid (175, 974) was employed for all calculations. It was confirmed that the optimized geometries corresponded to local minima on the potential energy surface and did not exhibit any imaginary frequency. Molecular electrostatic potential maps (MEPS) of the dimercaptobenzothiadiazole additives are calculated to examine the electron density in BBST and BOST.

## 3. Result and discussion

### 3.1 Properties of the additives

Fig. S3† shows the solubility and stability of both 10 wt% BBST and 10 wt% BOST in NBO. The results indicate that BOST was completely dissolved in NBO. After 30 days, 10 wt% BOST mixed oil is still in transparent oil liquid. On the other hand, flocculent precipitate was observed in NBO formulated with 10 wt% of BBST from days 0 until days 30. The good solubility and dispersivity of BOST in NBO is attributed to the presence of a long alkyl chain.^33^

Table 1 provides the density and viscosity for NBO, 10 wt% BBST, and 10 wt% BOST at three different temperatures (25 °C, 40 °C, and 100 °C). It is apparent that 10 wt% BBST (150.2) has a higher viscosity index (VI) compared to BOST (123.7). This indicated that BBST can form a thicker boundary lubrication film to protect the metal surface than that of BOST.^34^ The VI values are ranked as BOST < BBST < NBO. Fig. S4† depicted that the dynamic viscosity of NBO and 10 wt% additives decreased as the temperature increased.

**Table 1:** Density and viscosity of NBO, 10 wt% BBST and 10 wt% BOST

Lubricant		NBO	BBST	BOST
Density (g mL^−1^)	25 °C	0.9427	0.7872	0.8954
	40 °C	0.9423	0.7868	0.8939
	100 °C	0.9408	0.7848	0.8920
Dynamic viscosity (mPa s)	25 °C	31.50	44	42.00
	40 °C	22.50	23	21.50
	100 °C	5.50	5.00	4.50
Kinematic viscosity (mm^2^ s^−1^)	25 °C	36.79	55.89	46.91
	40 °C	26.28	29.23	24.05
	100 °C	6.42	3.82	2.80
Viscosity index		213.0	150.2	123.7

**Fig. 1:**
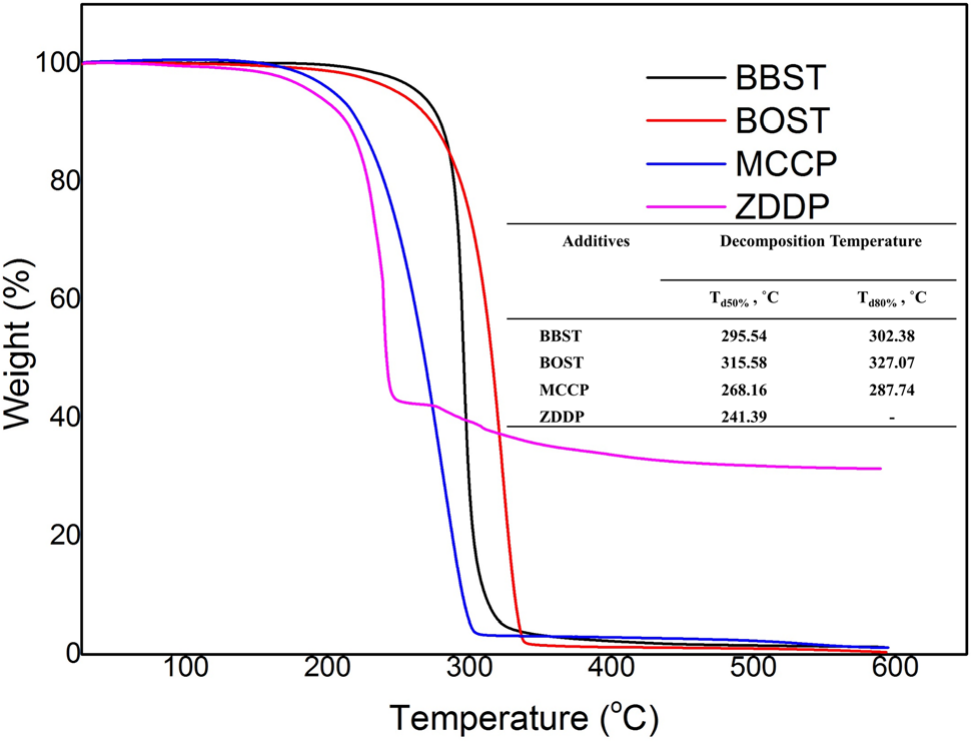
TGA curves of BBST, BOST, MCCP, and ZDDP in N_2_ atmosphere.

Fig. 1 shows the thermal stability of the BBST, BOST, MCCP, and ZDDP. The initial decomposition temperature (*T*_d_) of BBST, BOST, MCCP, and ZDDP are 284.69 °C, 281.19 °C, 217 °C, and 217 °C, respectively. The initial thermal degradation temperature of the BBST (284.69 °C) is higher than BOST (281.19 °C) might be due to the electronic delocalization and rigidity of the benzene ring in BBST. Lizarraga *et al.*^35^ reported that the urea and selenourea compounds containing aromatic substituents have higher initial decomposition temperature than those with aliphatic groups due to rigidity and electronic delocalization in the π orbitals of the aromatic ring. Besides, the initial thermal degradation temperature of the aromatic polyesters is higher than that of aliphatic polyesters presumably to the rigidity of the aromatic structures.^36^ However, the aromatic substituted polyesters are still decomposed faster than aliphatic substituted polyesters at temperature above 500 °C.

Furthermore, both BBST and BOST are more thermally stable than that of MCCP and ZDDP because of their high *T*_d_. Furthermore, the *T*_d_ for 50% and 80% weight loss of BBST (295.54 °C and 302.38 °C) is lower than BOST (310.36 °C and 321.34 °C), which indicates that BBST has lower thermal stability than that of BOST. Therefore, it is presumably that BBST decomposes faster than that of BOST and thus the S and N active elements have higher affinity to react with the metal surface and generate a tribofilm. Other studies^37^ also observed that additives with lower decomposition temperature has higher activity to react with the metal surface and generate a protective layer. Besides, the TG curve also showed that the BBST and BOST almost completely decomposed above 592 °C without any ash remaining. However, we observe that even after 500 °C, the ZDDP left around 30% solid ash due to its Zn content. Both BBST and BOST are more environmentally benign than ZDDP due to heavy metal-free. Besides, the ashless properties also prevent suspended formation and minimize the damage to the machine during operation.

**Fig. 2:**
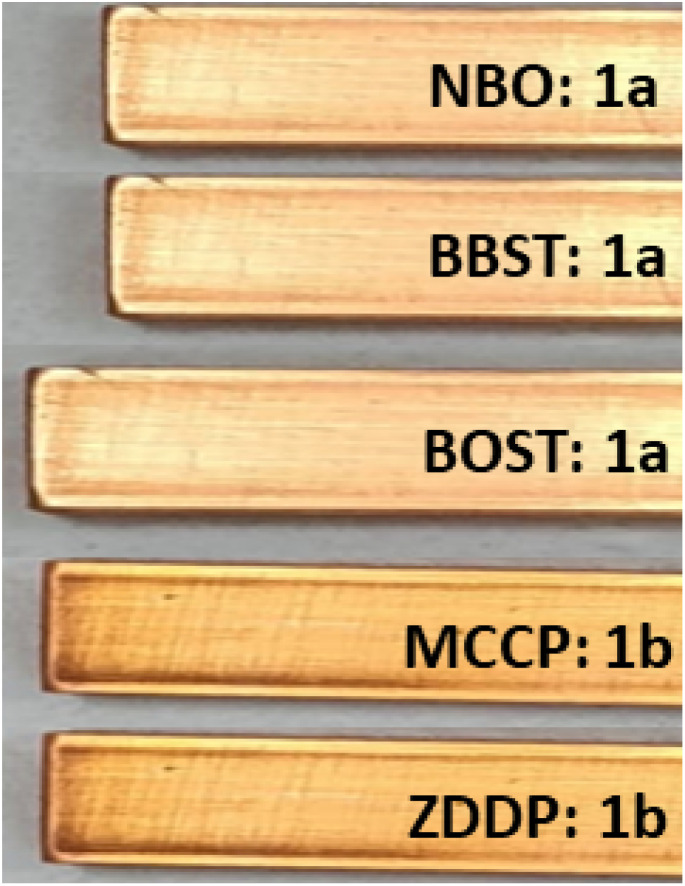
The photography of copper strips.

The corrosion grade of NBO, 10 wt% BBST, 10 wt% BOST, 10 wt% MCCP, and 10 wt% ZDDP are shown in Fig. 2. The copper strip of NBO appears as the freshly polished strip, which is grade 1a. The copper strips of BBST and BOST appear in light orange, which is also grade 1a. Nonetheless, the copper strips of MCCP and ZDDP exhibited mild discoloration (deep orange, 1b) after the test, which indicated that MCCP and ZDDP are corrosive to copper-based material.

The inhibiting mechanism of BBST and BOST can be ascribed to the adsorption of dimercaptobenzothiadiazole molecules on the metal surface.^38,39^ The adsorption mechanism of dimercaptobenzothiadiazole derivatives can be attributed to the interaction between the S and N elements present in the dimercaptobenzothiadiazole ring with the metal surfaces. The interaction between the lone pair electron in the N elements of the dimercaptobenzothiadiazole ring and the π electron in C=N double bond, known as P–π conjugation, plays a crucial role in enhancing the adsorption of dimercaptobenzothiadiazole derivatives onto the metal surface. Furthermore, the adsorption efficiency of BBST is higher than that of BOST due to the presence of benzene ring. The π bonds of the benzene ring in BBST can interact with the empty d-orbital of metal atoms, resulting in the formation of a more stable adsorption film. Conversely, the long carbon chains in BOST tend to align horizontally to cover more copper surface which protect metal surface from corrosion. Another study also observed that π electron in benzene rings, C=N bond, long carbon chains, S, and N elements can effectively formed a stable adsorption film to protect the metal surface.^38,40^

### 3.2 AW and tribological performance

**Fig. 3:**
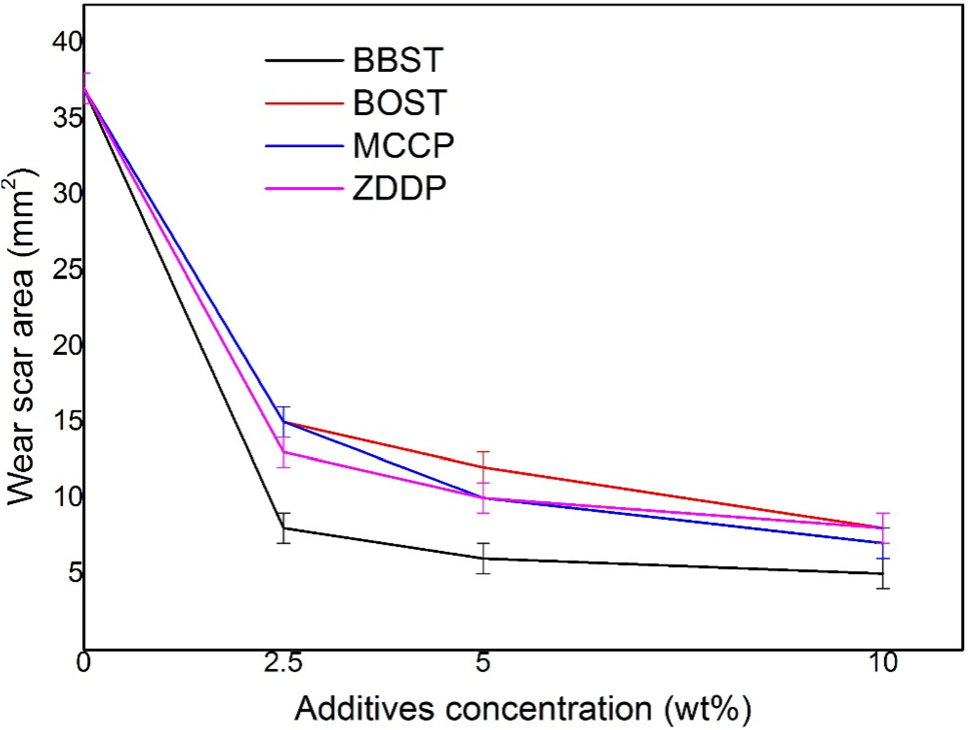
Variation of the WSA of steel roller with additives concentration.

Fig. 3 shows the wear scar area (WSA) obtained with base oil and all lubricant samples. It is observed that the addition of BBST, BOST, MCCP, and ZDDP resulted in decrease in the WSA of NBO at all added concentration. Furthermore, the AW performance of BBST was found to be superior to that of BOST at equivalent concentrations. The 10 wt% BBST was the best with 7.40 times of wear reduction with respect to the NBO. Besides, 10 wt% BOST showed 4.63 times of wear reduction and both 10 wt% MCCP and 10 wt% ZDDP presented 5.29 times and 4.63 times of wear reduction in comparison with the base stock. The wear results demonstrated that BBST has greatly improved the AW performance of the NBO compared to BOST, MCCP, and ZDDP, respectively. The overall AW performance of the additives can be ranked as BBST > MCCP > ZDDP > BOST.

As previously stated, the bonding between dimercaptobenzothiadiazole derivatives and metal surfaces occurs *via* electrostatic interaction between the S and N atoms in the dimercaptobenzothiadiazole ring and metal surfaces, resulting in the formation of coordinated bonds between S and N atoms with metal surfaces.^41,42^ In case of BBST, the presence of a benzene ring enhances its adsorption efficiency to metal surface than BOST due to the π-electron interactions. Therefore, the S and N element in the BBST has a higher affinity to react with the metal surface. Besides, as observed earlier, BBST also has lower thermal stability than BOST. Consequently, BBST decomposes faster than that of BOST and thus allows more S and N elements to coordinately react with the metal surface to produce a high-melting point and low-shear strength metal salt film.

**Fig. 4:**
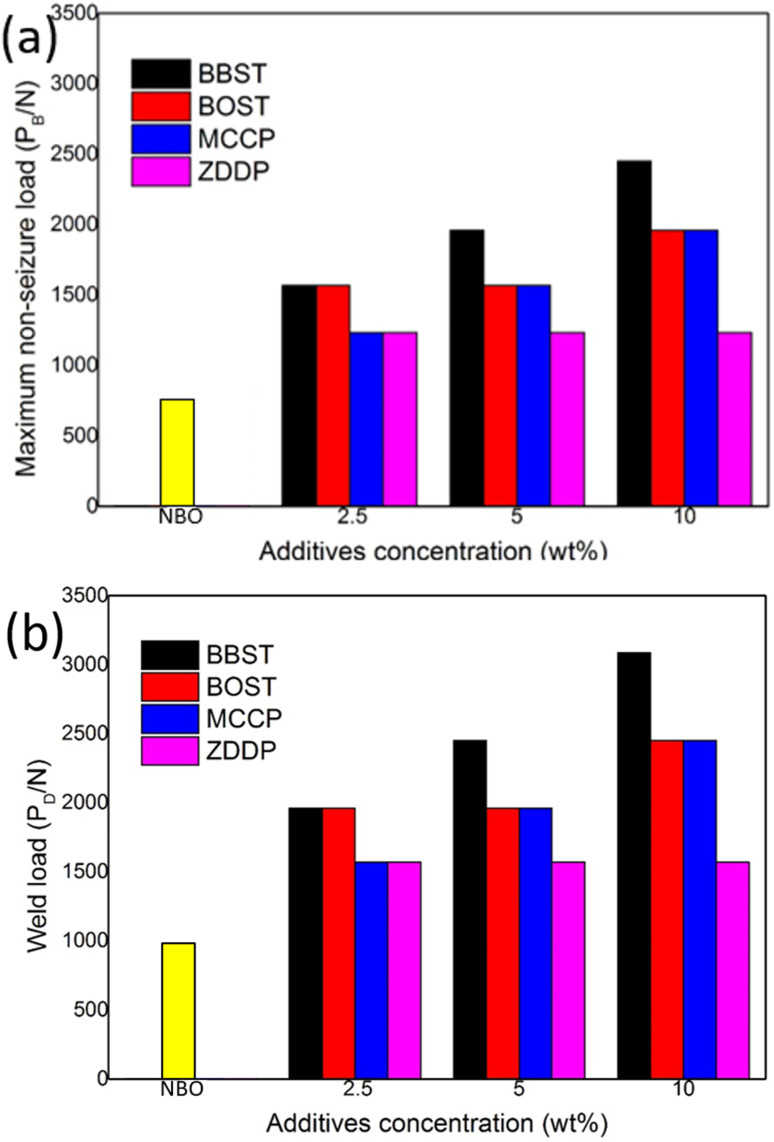
(a) *P*_B_ and (b) *P*_D_ value of BBST, BOST, MCCP, and ZDDP.

Fig. 4 shows the maximum non-seizure load (*P*_B_) and weld load (*P*_D_) of NBO with different concentration additives. Fig. 4 shows the initial *P*_B_ and *P*_D_ value of NBO are 785 N and 981 N, respectively. The results show that 10 wt% BBST has significantly improved the *P*_B_ value (2452 N) and *P*_D_ (3089 N) of NBO, which was 3.13 times that of NBO. While the *P*_B_ and *P*_D_ value of BOST reached 1961 N and 2452 N, which was 2.5 times that of NBO. This implies that both BBST and BOST can greatly enhance the load-carrying ability of NBO, and the *P*_B_ and *P*_D_ value of the NBO improved as the concentration of the additives increased. The BBST exhibited higher *P*_B_ and *P*_D_ than that of BOST.

The *P*_B_ and *P*_D_ value of the synthesized additives was also compared with commercial additives, such as MCCP and ZDDP. The *P*_B_ and *P*_D_ value of 10 wt% MCCP 1961 N and 2452 N, whereas 10 wt% ZDDP gives 1236 N and 1569 N, respectively. Both the BBST and BOST have higher EP performance than that of MCCP and ZDDP. The EP performance of the additives is ranked as BBST > BOST > MCCP > ZDDP.

As mentioned earlier, BBST has lower thermal stability than BOST. Thereby, it is presumably that the BBST is more easily decomposed than that of BOST and thus allow more S and N active elements to be absorbed on the metal surface to form an anti-seizure layer. The AW and EP performance of BBST and BOST is also in good agreement with their thermal stability.

### 3.3 Surface analysis

**Fig. 5:**
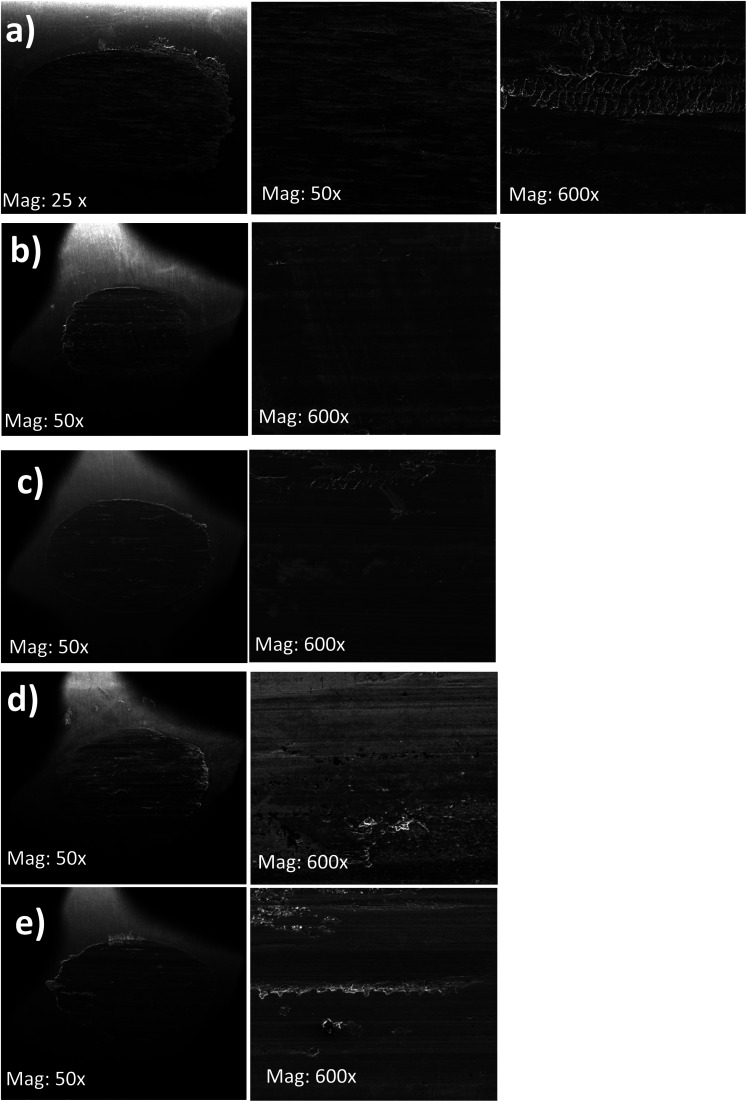
SEM images of the wear scars on the test cylindrical with 10 wt% (a) NBO, (b) BBST, (c) BOST, (d) MCCP, and (e) ZDDP in NBO.

The SEM images of the worn surfaces subjected to AW testing and lubricated with 10 wt% of BBST, 10 wt% BOST, 10 wt% MCCP and 10 wt% ZDDP are depicted in Fig. 5. It is found that the wear scar lubricated with NBO only consists of elements such as O, C, and Fe. This indicates that the larger wear scar observed in NBO is due to the absence of active elements, such as S, N, Cl, Zn, and P. It is observed that the wear scars of 10 wt% BBST and 10 wt% BOST are smoother and uniform than that of 10 wt% MCCP and 10 wt% ZDDP, respectively (Fig. 5). The worn scar of 10 wt% BBST (Fig. 5(a)) is much smaller, smoother, and uniform than lubrication with 10 wt% BOST (Fig. 5(b)). This revealed that the BBST can generate an effective boundary lubricating film to prevent the direct contact of test cylindrical with rotating ring compared to BOST, MCCP and ZDDP, which agrees with the excellent tribological performance of BBST.

Furthermore, the elemental composition of the worn surfaces lubricated with 10 wt% BBST and 10 wt% BOST shows the presence of S and N elements (Table 2 and Fig.S5†). The S and N contents deposited on the worn surfaces from BBST are 1.4 and 0.2 wt%, which is 70.0 and 66.7% higher than that of BOST. The worn surfaces of MCCP show the presence of Cl, whereas the ZDDP shows the presence of P, S, and Zn. The composition on the worn surfaces is in good agreement with their AW performance.

**Table 2:** Elements composition on the worn surfaces

Sample	EDX analysis (wt%)							
	C	Fe	O	S	N	Cl	Zn	P
NBO	8.5	90.1	1.4	—	—	—	—	—
BBST	9.7	86.7	2.0	1.4	0.2	—	—	—
BOST	7.7	90.0	1.6	0.6	0.1	—	—	—
MCCP	10.0	85.9	3.7	—	—	0.4	—	—
ZDDP	9.0	87.2	1.6	1.0	—	—	0.7	0.5

**Fig. 6:**
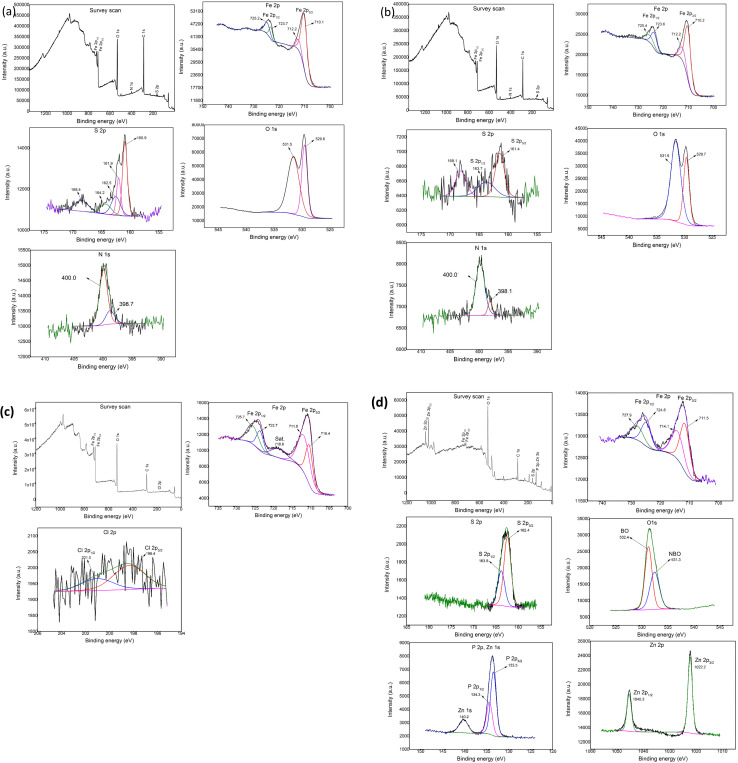
XPS of the wear scars on the test cylindrical with 10 wt% (a) BBST, (b) BOST, (c) MCCP, and (d) ZDDP in NBO.

Fig. 6(a) shows the results of the Fe 2p, S 2p, N 1s, and O 1s spectra obtained from the XPS analysis when lubricated with 10 wt% of BBST in NBO. From Fig. 6(a), the Fe 2p absorption peak at 710.1 and 723.7 eV belongs to Fe^2+^. In addition, the binding energy difference was 13.6 which is similar to reference iron sulfide (FeS).^43^ The Fe 2p peaks at 712.2 and 725.2 eV corresponded to the iron sulfate (FeSO_4_) and iron(iii) oxide (Fe_2_O_3_), and combined with the O 1s peak around 529.7 and 531.5 eV and S 2p peak around 168.4 eV, the existence of Fe_2_O_3_ and FeSO_4_ could be validated.^44^ The S 2p peak appeared at 160.9, 162.5, 161.9 and 164.2 eV correspond to sulfide.^43–45^ In the spectrum of N 1s, the peaks at 398.7 and 400.0 eV are attributed to C=N and Fe⋯N coordinate bonds.^44^ These results confirm that tribochemical reactions occurred when using BBST as lubricant additive.

Fig. 6(b) shows the results of the Fe 2p, S 2p, N 1s, and O 1s spectra obtained from the XPS analysis when lubricated with 10 wt% of BOST in NBO. The Fe 2p peak observed at 710.2 and 723.6 eV corresponds to the FeS bond, which is consistent with the S2p peak observed at 161.4 and 163.7 eV, respectively.^43^ The 712.2 and 725.4 eV attributed to Fe_2_O_3_ and FeSO_4_ (combined with O 1s at 529.7 and 531.6 eV and S 2p at 168.1 eV).^44^ The N 1s peak appeared at 398.1 and 400.0 eV belongs to C=N and Fe⋯N coordinate bonds.^44^ The present analysis assumes that the outstanding tribological performance of BBST and BOST in NBO is due to the generation of complex tribochemical products such as oxides, sulfide, and chemisorbed nitrogen compounds film during the tests.

The XPS analysis of the worn surfaces lubricated by MCCP shows the presence of iron(iii) chloride (FeCl_3_) and iron(iii) oxide-hydroxide (FeOOH) (Fig. 6(c)), whereas the ZDDP shows the presence of iron(iii) phosphate (FePO_4_), FeS, poly(thio)-phosphate chains, non-bridging oxygen (NBO), bridging oxygen (BO), P–O–C bonds, P–O–P bonds, and Zn_3_(PO_4_)_2_ (Fig. 6(d)), respectively.^30^ The difference in the tribology performance of dimercaptobenzothiadiazole derivatives, MCCP, and ZDDP is attributed to the chemical composition on the tribofilm.^4^ The melting point of the FeS tribofilm (∼1193 °C) is higher than that of FeCl_3_ (∼306 °C) and FePO_4_ (∼250 °C) tribofilm. Consequently, the sulfur-based additives have higher weld preventing and protective film properties than that of chlorine- and phosphorus-based additives and thus gives excellent EP and AW performance.

### 3.4 Tribofilm formation and mechanism

Based on the XPS spectra, the chemical composition of the worn surfaces for BBST and BOST can be ranked as follows: FeSO_4_ > Fe_2_O_3_ > FeS > Fe⋯N coordinate bonds > C=N. The high amount of O elements content is probably due to the direct surface reactions and oxygen diffusion process.^30,46^ As mentioned in our previous studies,^30^ the nascent iron surface will react with sulfur and oxygen to form an oxide interlayer, such as FeSO_4_ and Fe_2_O_3_. Furthermore, the base oil might also oxidized during the wear process to generated Fe_2_O_3_.^47^ Besides, during the rubbing process, oxygen will diffuse through the tribofilm and react with the iron substrates to generate the oxide interlayer. The top layer tribofilm might further oxidized to generate FeSO_4_ during the storage after the tribo-test.^48^

Based on the EDX and XPS, it can be inferred that the π-electrons of the benzene ring and the lone pairs of N and S elements will electrostatically interact or physisorbed onto the iron surface. Subsequently, the N and S elements of the dimercaptobenzothiadiazole ring will undergo chemisorption with the metal surface to form a complex boundary film consisting of FeS, FeSO_4_, Fe_2_O_3_ and Fe⋯N coordinate bonds. This boundary lubrication film minimizes wear and prevents welding between the metal surfaces. It is presumably FeS and Fe_2_O_3_ are the main contributor to the excellent tribology performance due to their high melting point (>1193 °C).

**Fig. 7:**
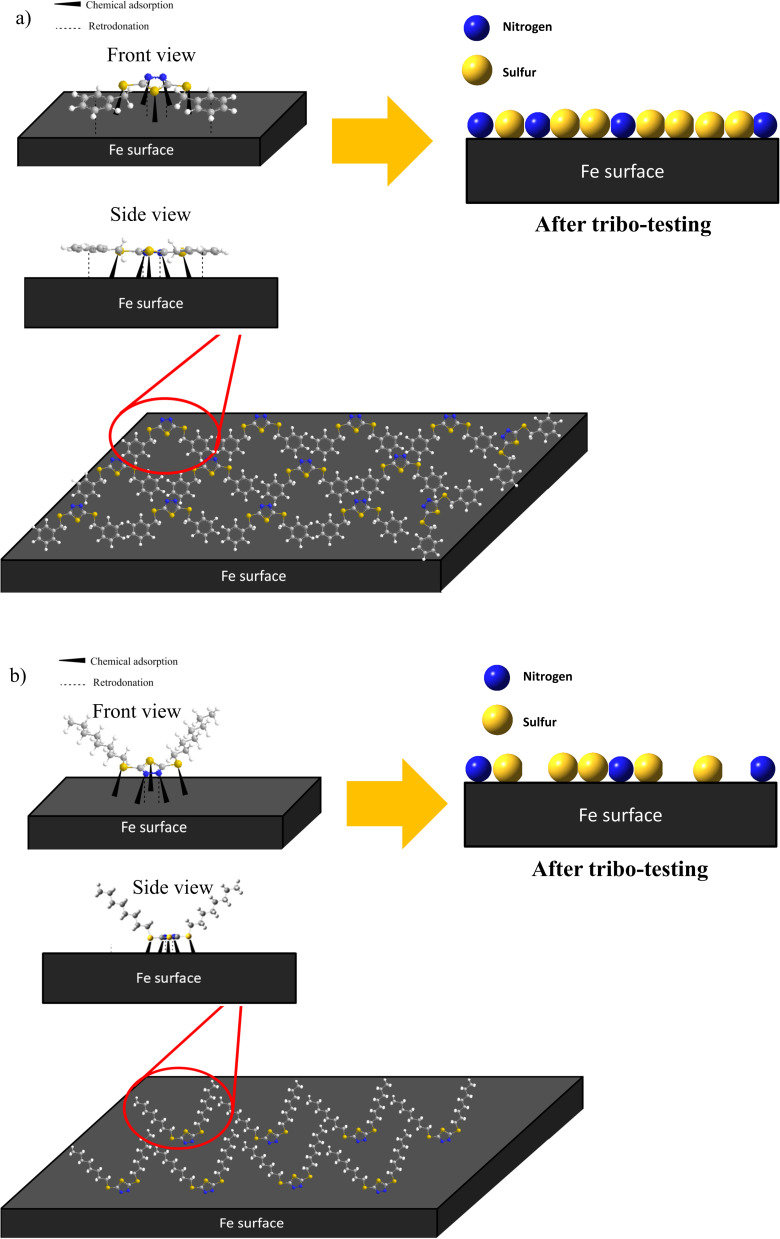
Illustration of (a) BBST and (b) BOST on metal surface.

Furthermore, the difference in the tribology performance of BBST and BOST is owing to the chemical structure. The MEPS displayed that the BBST has higher electron density in the overall structure due to the larger conjugated system from benzene ring to dimercaptobenzothiadiazole ring (Fig S6(a)†). Consequence, the π-electrons of the benzene ring in BBST could establish a non-covalent interaction with the vacant d-orbital of the iron surface to creates a donor–acceptor interaction that promotes the interactions between BBST and metal surface. The strong non-covalent interaction between the BBST and metal surface can create favorable conditions for the formation of coordination bonds, thus more S and N element from the dimercaptobenzothiadiazole can react with the metal surface to generate a metal salt tribofilm such as FeS, FeSO_4_, and Fe⋯N coordinate bonds. On the contrary, the hydrocarbon chains in BOST have less electron density as compared with BBST, and thus it has least interaction with the metal surface (Fig S6(b)†). Furthermore, the hydrocarbon chains in BOST may align horizontally to the metal surface, which will reduce and block other BOST molecules from adsorbing on the metal surface. Moreover, the BBST decomposes faster than BOST and thus allows more S and N active elements to adsorb on the metal surface and form a tribofilm. The EP and AW performance of BBST and BOST is in good agreement with their thermal stability. Therefore, both the S and N elements play a significant role in non-corrosive, AW, and EP performance. The adsorption of BBST and BOST on metal surface is illustrated in Fig. 7.

To examine the efficiency of the synthesized additives, we compare our additives with the previously reported additives which also use NBO as a base stock (Table 3). To the best of our knowledge, only three reports used NBO as base stock.^30,49,50^ Nano-TiO_2_ showed the lowest WSD at 0.07 wt%, which improved 1.03 times of wear reduction of the NBO.^49^ The addition of 0.1 wt% of graphite nanoflakes improved 2.5 times of wear reduction of the NBO but highly corrosive (corrosion grade 2c) to copper-based materials.^50^ Besides, our previous works^30^ found that the dioctyl disulfides and dibenzyl disulfides additives are non-corrosive and possess excellent EP and AW performance in the NBO. In this work, the dimercaptobenzothiadiazole-based additives, such as BBST are found to be ashless, non-corrosive, and exhibit excellent EP and AW performance compared to toxic conventional additives, such as MCCP and ZDDP. This indicated that BBST shows promising potential as an alternative EP and AW additives to replace MCCP and ZDDP. In general, NBO is a widely used base oil for industrial applications, such as insulating oil, compressor oil, and MWFs, *etc*^29^. Therefore, the exploration of the EP and AW additives in NBO is highly desired.

**Table 3:** Tribological performance based on NBO as base stock

Entry	Additives	EP		AW (Wear reduction times)	Corrosion grade	Ref.
		*P*_B_ (N)	*P*_D_ (N)			
1	0.07 wt% nano-TiO_2_	—	—	1.03	1a	46
2	0.1 wt% graphite nanoflakes	—		2.50	2c	47
3	10 wt% dioctyl disulfides	1569	1961	4.63	1a	30
4	10 wt% dibenzyl disulfides	1961	2452	6.20	1a	30
5	10 wt% MCCP	1961	2452	5.29	1b	This work
6	10 wt% ZDDP	1236	1569	4.63	1b	This work
7	10 wt% BOST	1961	2452	4.63	1a	This work
**8**	**10 wt% BBST**	**2452**	**3089**	**7.40**	**1a**	**This work**

## 4. Conclusion

In summary, the conclusions are given below:

(1) The synthesized dimercaptobenzothiadiazole derivatives, BBST and BOST consist of three sulfur atoms that are non-corrosive and ashless.

(2) The EP and AW of BBST are 3.13 and 7.40 times better than NBO at 10 wt%. The performance of BBST is also better than BOST, MCCP, and ZDDP.

(3) The outstanding tribology performances of BBST compared to BOST is due to the interaction of S, N, and π-electrons of benzene ring with the metal, and fast decomposition.

(4) BBST and BOST show great promise to replace commercial additives, such as MCCP and ZDDP.

(5) The formation of FeS and Fe_2_O_3_ under AW conditions provides excellent boundary lubrication films.

## Author contributions

Chiu Ling Ong: data curation, writing – original draft preparation. Yew Chong Lai: investigation. Thorsten Heidelberg: supervision and editing. Wai Kit Tang: visualization and software. Vannajan Sanghiran Lee: software. Nader Ghaffari Khaligh: supervision, conceptualization, and reviewing. Joon Ching Juan: supervision, conceptualization, reviewing and editing.

## Conflicts of interest

There are no conflicts to declare.

## Supplementary Material

RA-013-D3RA05692A-s001
