# Supplementary material for: Highly effective ashless and non-corrosive dimercaptobenzothiadiazole as multifunctional lubricant additives in naphthenic base oil
Source: RSC Adv. 2023 Oct 19;13(44):30733–42. doi: 10.1039/d3ra05692a (PMC10585983; doi:10.1039/d3ra05692a)
Supplement: RA-013-D3RA05692A-s001 [file RA-013-D3RA05692A-s001.pdf]

Electronic Supplementary Material (ESI) for RSC Advances.
This journal is © The Royal Society of Chemistry 2023

Supplementary Data

List of Figure

Fig. S1. The (a) ^{1}H NMR, (b) ^{13}C NMR and (c) GCMS spectral data of BBST.

Fig. S2. The (a) ^{1}H NMR, (b) ^{13}C NMR and (c) GCMS spectral data of BOST.

Fig. S3. Samples photo: (a) NBO, (b) NBO + 10 wt% BBST, and (c) NBO + 10 wt% BOST.

Fig. S4. Average dynamic viscosity as a function of temperature for NBO, 10 wt% BBST and 10 wt% BOST.

Fig. S5. Elemental composition of the wear scars on the test cylindrical with (a) NBO, and 10 wt% (b) BBST, (c) BOST, (d) MCCP, and (e) ZDDP in NBO.

Fig. S6. MEPS of (a) BBST and (b) BOST.

Fig. S7. XPS of the wear scars lubricated with NBO alone.

BBST. White solid. 90% Isolated yields. mp 83-85 ˚C. **^{1}H NMR (600 MHz, $CDCl_3$):** δ 7.40 (d, *J* = 7.3 Hz,
4H), 7.33 (t, *J* = 7.2 Hz, 4H), 7.29 (t, *J* = 7.2 Hz, 4H), 4.50 (s, 4H) ppm. **^{13}C NMR (600 MHz, $CDCl_3$):** δ 164.93, 135.92, 129.34, 128.92, 128.10, 38.61 ppm. **MS (EI, 70 eV)** m/z 330.

Fig. S1. The (a) ^{1}H NMR, (b) ^{13}C NMR and (c) GCMS spectral data of BBST.

(a)

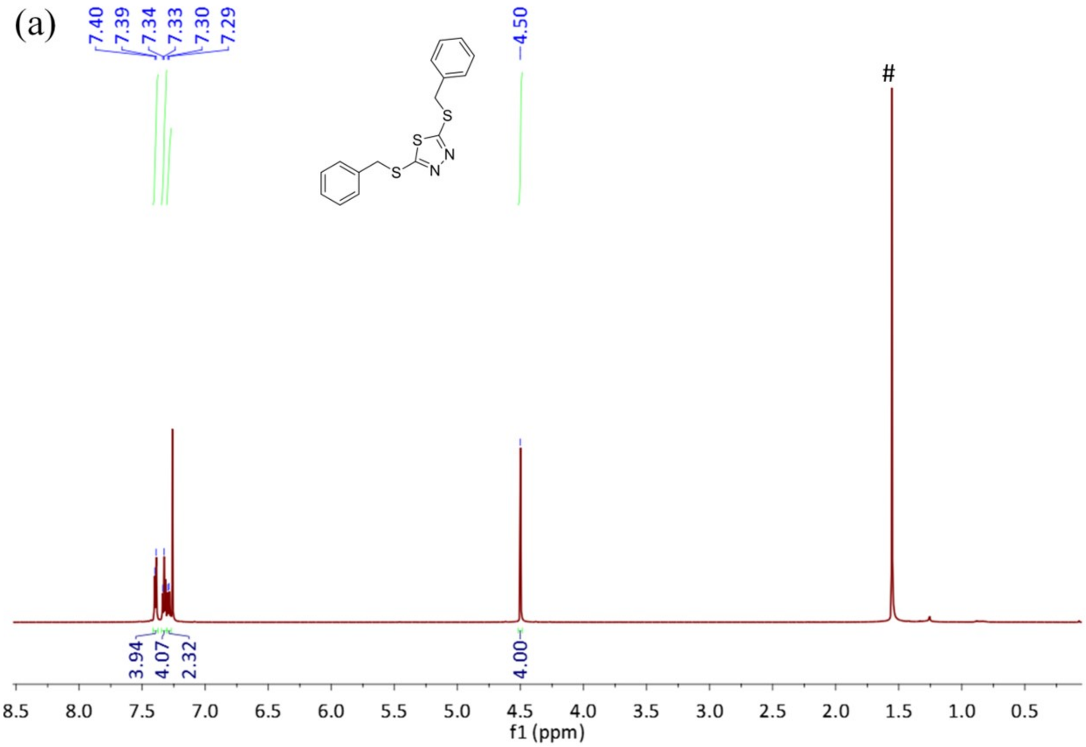

The resonance marked with # is originating from water.

(b)

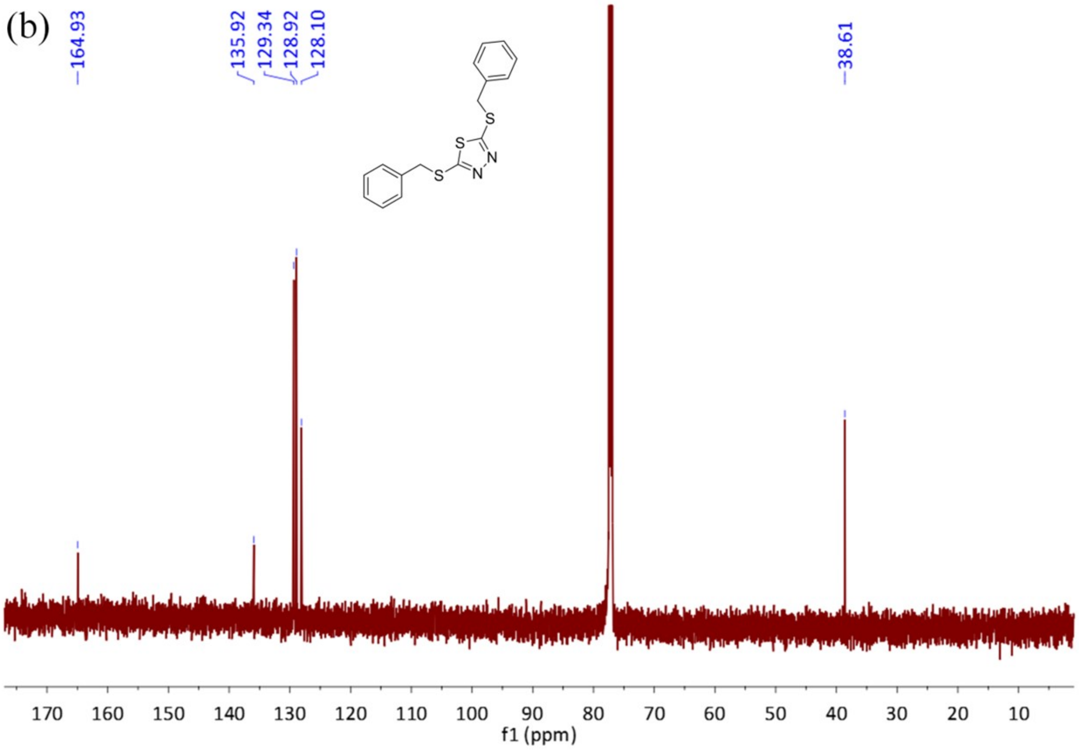
164.93
135.92
129.34
128.92
128.10
38.61
S
S
S
N
N
170 160 150 140 130 120 110 100 90 80 70 60 50 40 30 20 10
f1 (ppm)

(c)

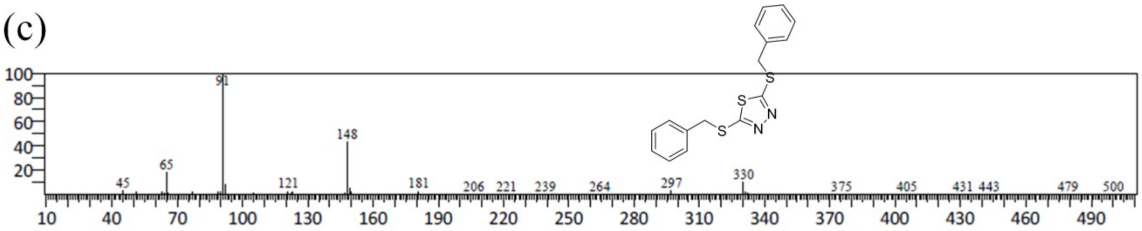
100
80
60
40
20
91
148
65
45
121
181
206
221
239
264
297
330
375
405
431
443
479
500
S
S
S
N
N
10 40 70 100 130 160 190 220 250 280 310 340 370 400 430 460 490

BOST. Transparent liquid. 97 % Isolated yields. **^{1}H NMR (600 MHz, $CDCl_3$):** δ 3.26 (t, *J* = 7.4 Hz, 2H), 1.78 (quint, *J* = 7.5 Hz, 4H), 1.45 - 1.26 (m, 10H), 0.87 (dd, *J* = 7.1 and 6.8 Hz, 3H) ppm. **^{13}C NMR (600 MHz, $CDCl_3$):** δ 165.41, 34.52, 31.90, 29.35, 29.26, 29.16, 28.85, 22.76, 14.21 ppm. **MS (EI):** m/z calcd for $C_{18}H_{34}N_2S_3Na^+$ $[M+Na]^+$: 397.1777; found: 397.1655; m/z calcd for $C_{36}H_{68}N_4S_6Na^+$ $[2M+Na]^+$: 771.3661 ; found: 771.3575.

Fig. S2. The (a) ^{1}H NMR, (b) ^{13}C NMR and (c) GCMS spectral data of BOST.

(a)

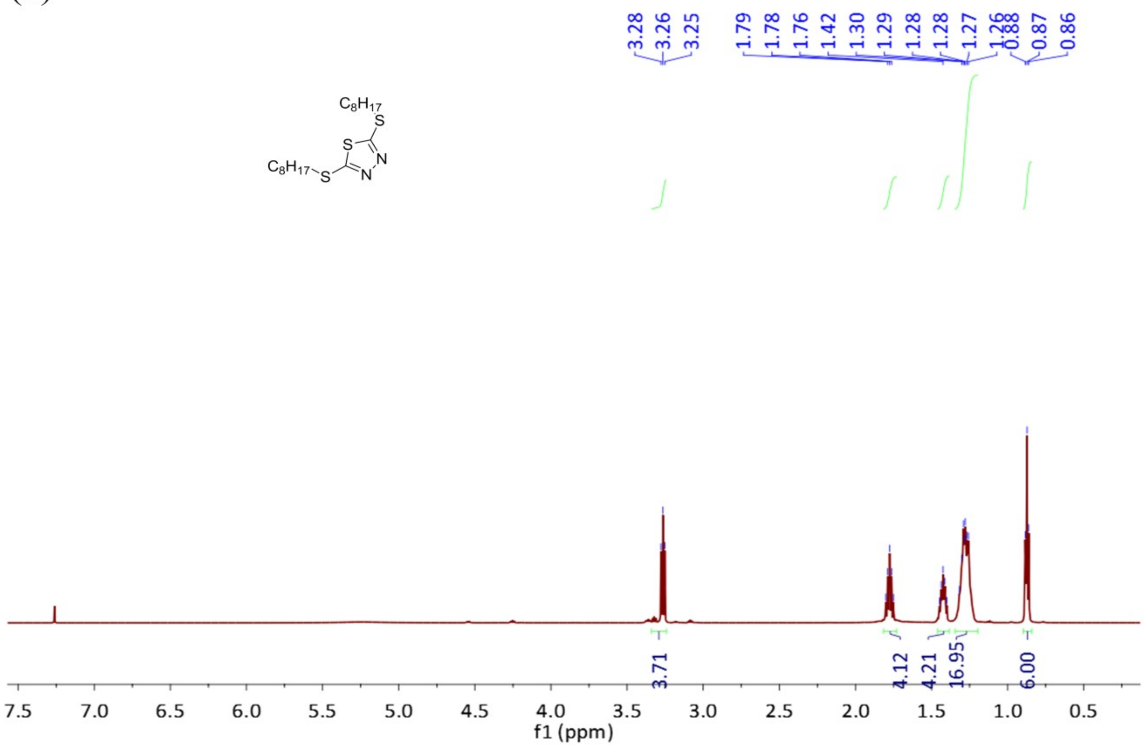

(b)

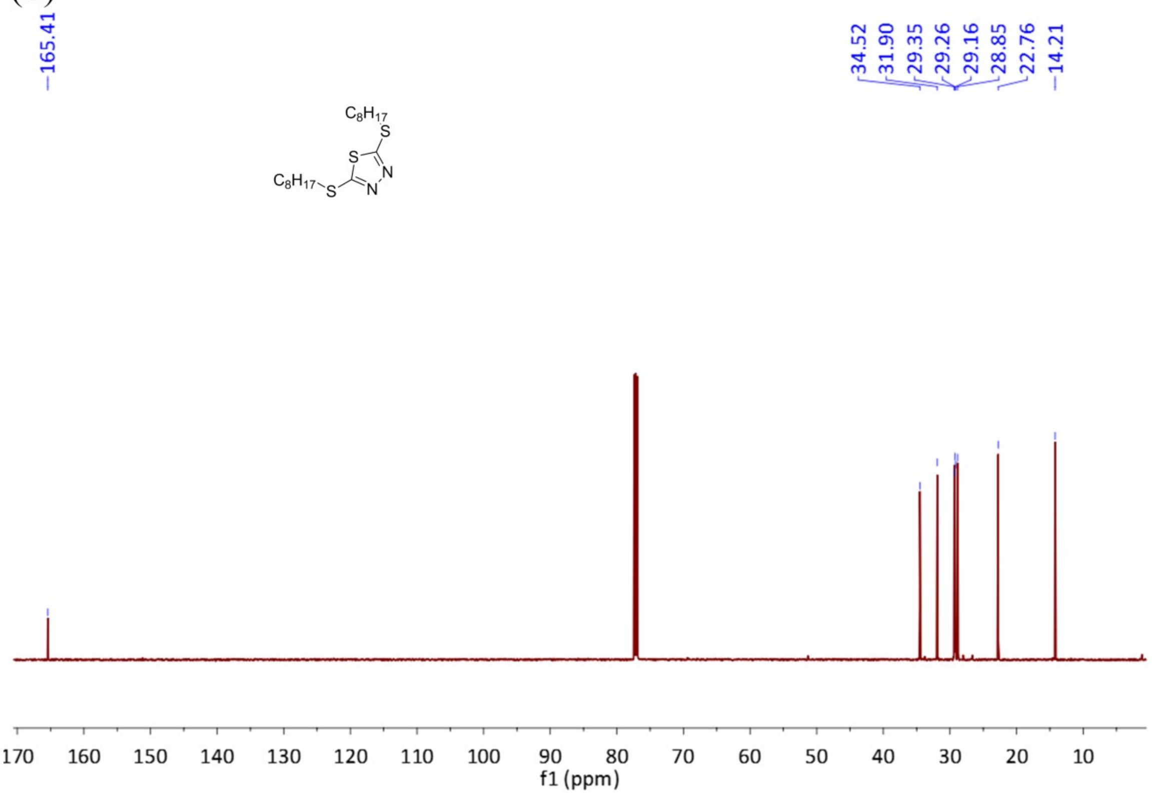

165.41
34.52
31.90
29.35
29.26
29.16
28.85
22.76
14.21
C8H17
S
S
N
N
C8H17
S
f1 (ppm)

(c)

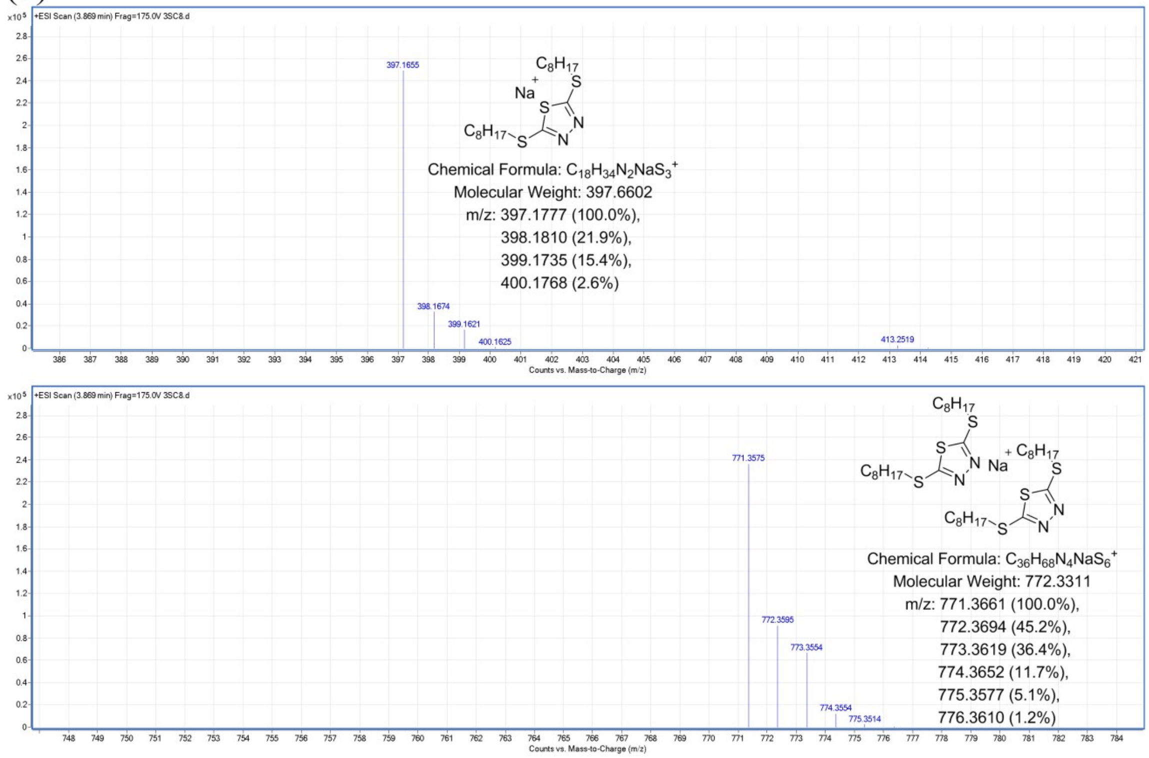

+ESI Scan (3.869 min) Frag=175.0V 3SC8.d
397.1655
398.1674
399.1621
400.1625
413.2519
Chemical Formula: C18H34N2NaS3+
Molecular Weight: 397.6602
m/z: 397.1777 (100.0%),
398.1810 (21.9%),
399.1735 (15.4%),
400.1768 (2.6%)
Counts vs. Mass-to-Charge (m/z)
+ESI Scan (3.869 min) Frag=175.0V 3SC8.d
771.3575
772.3595
773.3554
774.3554
775.3514
Chemical Formula: C36H68N4NaS6+
Molecular Weight: 772.3311
m/z: 771.3661 (100.0%),
772.3694 (45.2%),
773.3619 (36.4%),
774.3652 (11.7%),
775.3577 (5.1%),
776.3610 (1.2%)
Counts vs. Mass-to-Charge (m/z)

Fig. S3. Samples photo: (a) NBO, (b) NBO + 10 wt% BBST, and (c) NBO + 10 wt% BOST.

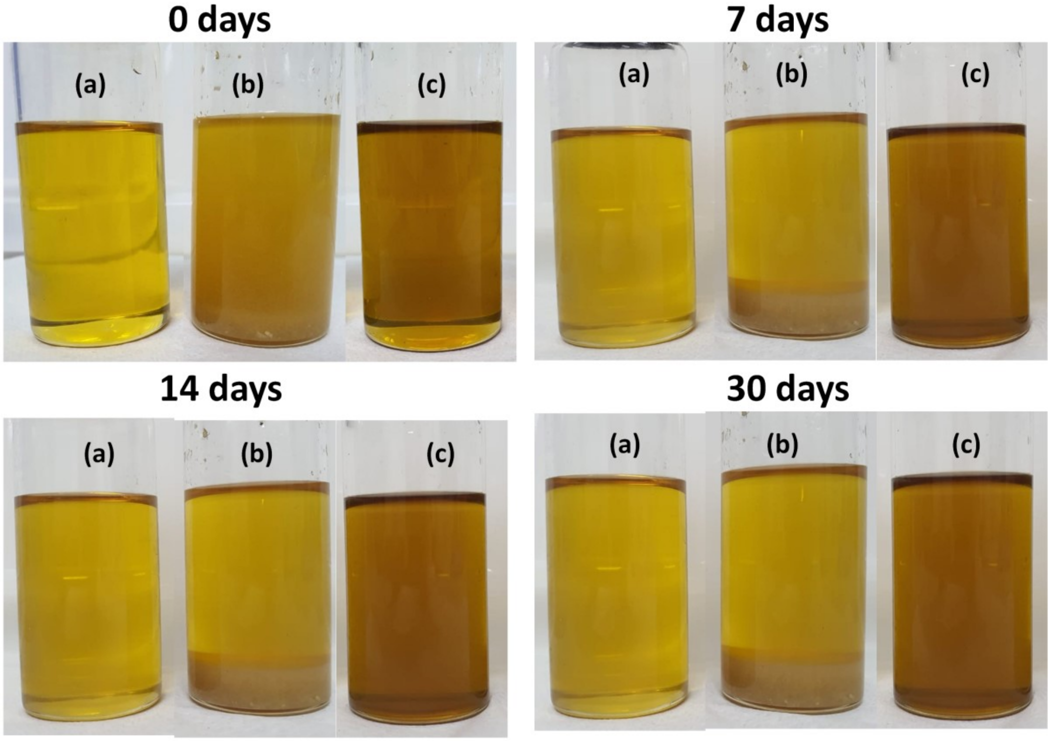

Fig. S4. Average dynamic viscosity as a function of temperature for NBO, 10 wt% BBST and 10 wt% BOST.

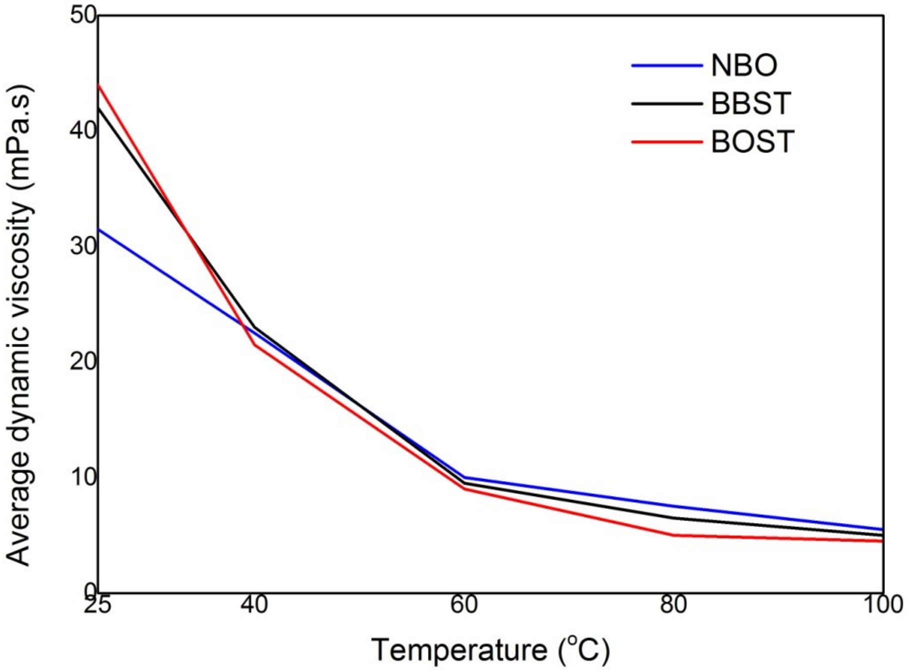

Fig. S5. Elemental composition of the wear scars on the test cylindrical with (a) NBO, and 10 wt% (b) BBST, (c) BOST, (d) MCCP, and (e) ZDDP in NBO.

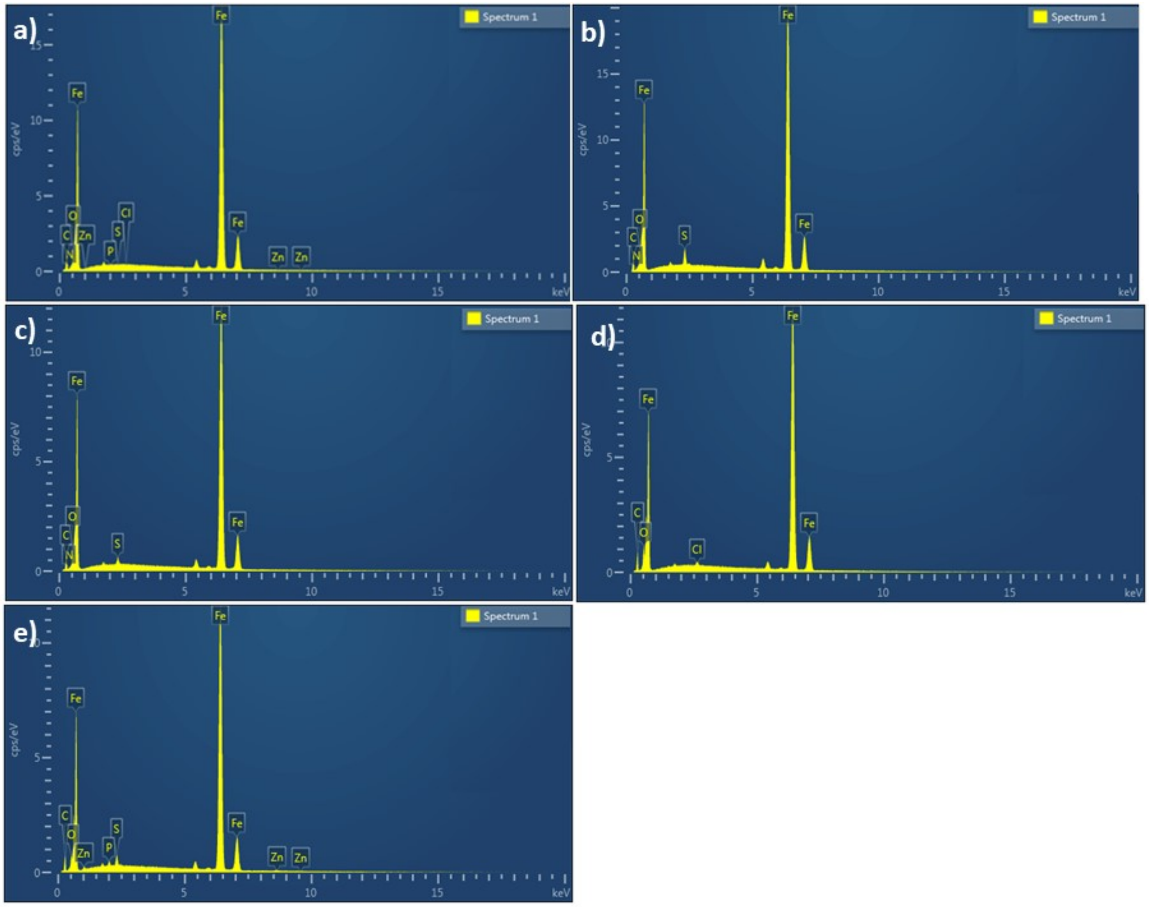

Fig. S6. MEPS of (a) BBST and (b) BOST.

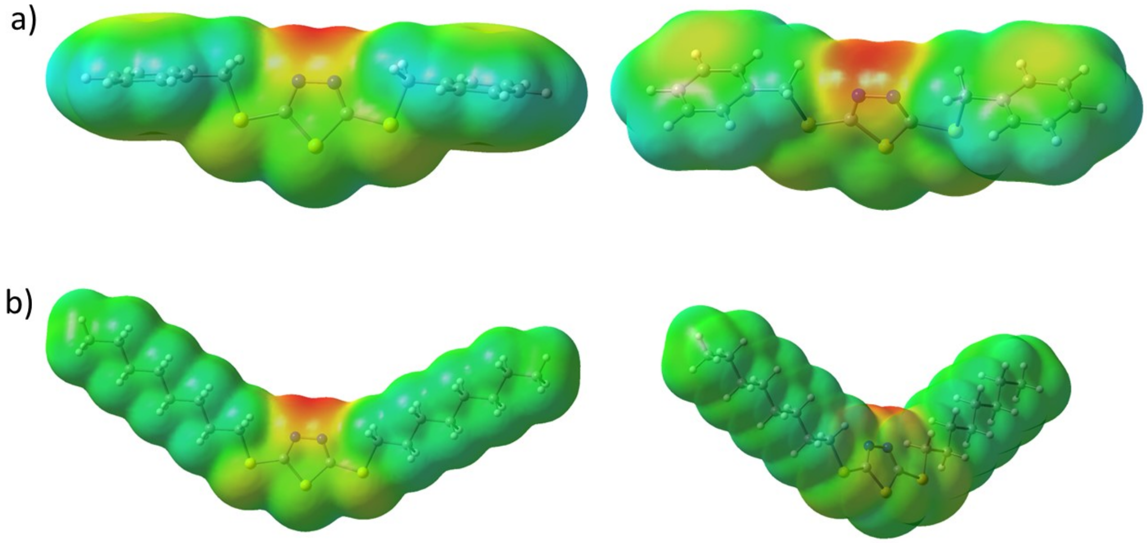

Fig. S7. XPS of the wear scars lubricated with NBO alone.

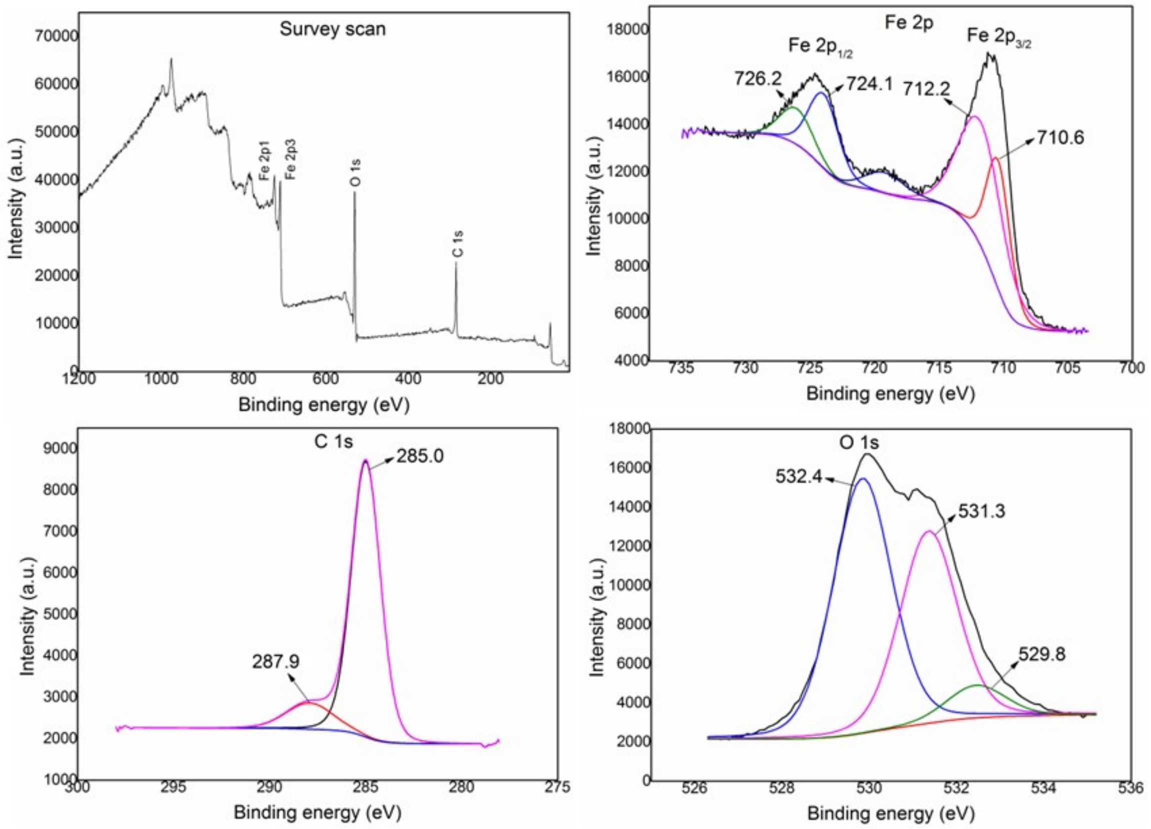

List of Table

Table S1 Chemicals composition on the worn surfaces.

Sample	XPS analysis Narrow scan (at %)					
	S 2p		O 1s		N 1s	
	FeS	$FeSO_4$	Fe_2O_3	$FeSO_4$	C=N	Fe···N
BBST	87.9	12.1	38.2	61.8	21.1	78.9
BOST	70.4	29.6	32.7	67.3	11.1	88.9
